# Observational study of the effects of upper respiratory tract infection on hydration status

**DOI:** 10.1186/s40248-019-0200-9

**Published:** 2019-10-31

**Authors:** Ronald Eccles, Pascal Mallefet

**Affiliations:** 1 Cardiff School of Biosciences, Cardiff University, Sir Martin Evans Building, Museum Avenue, Cardiff, CF10 3AX UK; 2 GSK Consumer Healthcare Company, Route de l’Etraz 2, 1260 Nyon, Switzerland

**Keywords:** Dehydration, Upper respiratory tract infections, Common cold, Human influenza

## Abstract

**Background:**

A frequent treatment recommendation during acute respiratory infection is to increase fluid intake. This is the first study to investigate whether upper respiratory tract infections (URTIs) such as common cold can lead to dehydration, as commonly believed by the public.

**Methods:**

This was an exploratory, noninterventional, observational, single-center study. Subjects made 2 visits to a UK study center for assessments of dehydration, once during URTI and then 2–3 weeks later when fully recovered. The primary endpoint was a comparison of serum osmolality during vs after URTI. Complete blood count, serum urea, serum electrolytes, urine parameters (eg, osmolality, specific gravity, color), body weight/BMI, subjective assessment of thirst, and physician assessment of dehydration were additional outcomes. Only descriptive statistics and shift tables were used.

**Results:**

Fifty-five otherwise healthy adults with moderate to severe URTI of < 120 h in duration were enrolled (63.6% female, 94.5% white, mean [SD] age 21.0 [6.8] years). There was no evidence of dehydration based on serum osmolality (mean [SD] 287.63 [4.83] mosm/kg during URTI; 288.60 [5.99] mosm/kg after recovery). With only a few exceptions, complete blood count, serum urea, serum electrolytes, urine specific gravity, urine color, and physician ratings of hydration remained stable. Body weight decreased > 1% in 34.0% of subjects and increased > 1% in 17.0% between visits, with similar changes in BMI. Urine osmolality varied: 14 subjects showed a decrease and 5 showed an increase, resulting in a higher mean [SD] urine osmolality during URTI (700.50 [231.59] vs 618.47 [320.29] mosm/kg). Subjects perceived greater thirst during URTI.

**Conclusions:**

In this pilot observational study, we found no evidence that URTIs such as common cold are associated with dehydration, contrary to popular belief.

## Background

Folklore holds that upper respiratory tract infections (URTIs) such as common cold can lead to dehydration, but there are no solid, clinical data in the medical literature to substantiate this view. Regardless, health authorities and reputable medical establishments such as the National Health Service in the United Kingdom and the Mayo Clinic in the United States all recommend that persons suffering from the common cold should stay hydrated [1, 2]. This perception may have originated from early days when URTIs were accompanied by severe fevers. Hypothetically, persons with high fevers may lose fluids through sweating, and people may fail to adequately replace these lost fluids if they reduce eating/drinking because of loss of appetite.

In healthy individuals, even mild dehydration can slow cognitive decision-making, increase fatigue and headache, and impair memory, mood, alertness, concentration, physical performance, and endurance [3–8]. Severe dehydration can result in renal impairment, cardiovascular dysfunction, weakness, confusion, headache, nausea, malaise, fatigue, and sleepiness [6, 9]. However, no single “gold standard” method exists that is easy to use, accurate, precise, rapid, and comprehensive for assessing dehydration, although several techniques have been described such as patient history and physical examination, imaging techniques, and laboratory testing [10, 11].

This is the first pilot study to investigate whether there is evidence of dehydration during a self-diagnosed URTI such as common cold. As the US Institute of Medicine considers serum osmolality to be a primary indicator of hydration status [12], dehydration was identified in this study by comparing serum as well as urine osmolality of subjects with moderate to severe URTIs with serum and urine osmolality in the same subjects after recovery.

## Methods

### Study design and procedures

This non-interventional, observational, within-subject study was conducted from November 16, 2011 through April 3, 2012 at Common Cold Centre & Healthcare Clinical Trials, Cardiff University, Cardiff, UK (GSK protocol C6790895). After confirmation of eligibility, subjects visited the study site twice: once within 120 h of URTI symptom onset (Visit 1) and again 14 to 21 days later after they felt fully recovered (Visit 2). Subjects thereby served as their own controls to minimize the variability inherent in each individual’s levels of hydration. At both visits, investigators assessed the participants for the presence of URTI symptoms and performed urine and blood sampling, body weight measurement, and calculation of body mass index (BMI).

At both visits, participants provided subjective ratings of thirst, and the physician performed a hydration assessment and evaluated any adverse events. Subjective ratings of thirst were based on a visual analog scale (VAS) ranging from 0 = no thirst at all to 100 = worst thirst ever experienced. Physician ratings of hydration were based on skin turgor, dryness of tongue and mucous membranes, and general appearance, each rated on a scale of 0 = no evidence of dehydration, 1 = some dehydration, 2 = moderate dehydration, and 3 = severe dehydration.

All subjects provided informed consent prior to participation. The protocol was reviewed and approved by an independent ethics committee, and the study was conducted in accordance with requirements specified in the Declaration of Helsinki.

### Study population

Adult (≥18 years of age) participants were required to have symptoms of URTI for < 120 h prior to Visit 1; there was no requirement to have fever at inclusion. At Visit 1, at least 3 of 6 URTI symptoms (nasal congestion, sneezing, sore throat, chilliness, malaise, and nasal discharge) had to be moderate to severe, defined as a rating of 2 or 3 on a visual rating scale of 0 = none, 1 = mild, 2 = moderate, and 3 = severe. At Visit 2, subjects could not have a score > 1 on more than 2 of the URTI symptom visual rating scales to be eligible to continue in the study. If ineligible, the subject was permitted to repeat Visit 2 as long as the repeat visit was still within 14 to 21 days of Visit 1. Participants had to otherwise be in good general health with no clinically significant or relevant abnormalities in the medical history or physical examination, in the opinion of the investigator.

Screened individuals were excluded if the current illness was the first cold they had ever experienced. Additional exclusion criteria included pregnancy or breastfeeding, history of perennial allergic rhinitis or other chronic respiratory disease considered clinically significant by the investigator, and concurrent illness or medical history that could potentially affect serum or urinary hydration measurement (eg, renal disease). Potential subjects were also excluded if they were using medications that may cause dry mouth or affect hydration, or had used medications to treat URTI symptoms prior to Visit 1, including antibiotics in the last 7 days, analgesics/antipyretics in the last 24 h, decongestants in the last 12 h, or an antitussive or medicated lozenge or throat spray in the last 8 h. Use of these medications was also prohibited during the study period. Additional exclusion criteria included alcohol consumption or strenuous activity/exercise in the 12 h prior to either visit, and air travel > 3 h in duration during the 24 h prior to either visit. Anyone who had participated in another clinical study, received an investigational drug within 30 days of screening, or was an employee or family member of an employee of the sponsor or study site was prohibited from enrolling.

### Study outcomes

Evidence of dehydration was defined as a difference in 1 or more measures of hydration between Visit 1 (with URTI) compared with Visit 2 (after recovery at 14 to 21 days after Visit 1). The primary endpoint was a comparison of serum osmolality during moderate to severe URTIs with the serum osmolality of the same subjects when they had recovered. The following additional outcomes measures were explored: hematocrit, serum urea and electrolytes (sodium, potassium, chloride, and bicarbonate), complete blood count, urine specific gravity, urine osmolality, urine color, body weight and BMI, and subjective assessment of thirst. Table 1 defines percentage changes considered to be of clinical interest, with change being determined as Visit 2 (after recovery) minus Visit 1 (with URTI).

**Table 1:** Changes in dehydration-related outcomes considered to be of clinical interest between study visits

Outcome	Change of Interest
Serum osmolality	> 5%
Urine specific gravity	> 5%
Urine osmolality	> 10%
Urine color	> 2 colors
Hematocrit	> 5%
Serum urea and electrolytes	
Sodium	> 5%
Potassium	> 5%
Bicarbonate	> 5%
Urea	> 10%
Chloride	> 5%
Complete blood count	
Hemoglobin	> 5%
Red blood cell count	> 5%
Hematocrit	> 5%
Mean cell volume	> 5%
Mean cell hemoglobin	> 5%
Mean cell hemoglobin concentration	> 5%
White cell count	> 10%
Platelet count	> 10%
Body weight	> 1%
BMI	> 1%
Subjective assessments of thirst and hydration	
Subject ratings^a^	> 5%
Physician ratings^b^	
Skin	> 1 point
Tongue	> 1 point
Mucous membrane	> 1 point
General appearance	> 1 point

^a^Rated by subject on a visual analog scale ranging from 0 = no thirst at all to 100 = worst thirst ever experienced

^b^Rated by physician on a scale of 0 = no evidence of dehydration, 1 = some dehydration, 2 = moderate dehydration, and 3 = severe dehydration

*BMI* body mass index, *URTI* upper respiratory tract infection

### Laboratory analyses

Blood samples were divided into hematology and biochemistry/osmolality samples. The hematology samples were stored and shipped to the laboratory (Simbec Research, Ltd., Merthyr Tydfil, UK) at temperatures between 4 °C and 25 °C and were analyzed within 48 to 72 h. Hematology blood samples were analyzed using a Siemens Advia 2120 or Siemens Advia 120 (Siemens Healthcare GmbH, Erlangen, Germany) hematology testing system.

The biochemistry/osmolality samples were allowed to stand for 30 min and then centrifuged at x2000g for 10 min before 2 mL of serum was extracted and shipped at approximately 4 °C to the clinical laboratory (Simbec Research, Ltd.), where they were analyzed within 1 day. Biochemistry blood samples were analyzed using the Roche Modular Analytics System (F. Hoffmann-La Roche, Basel, Switzerland).

Urine samples were stored for up to 7 days at 4 °C, and then were shipped at the same temperature to the clinical laboratory (Simbec Research, Ltd.). Urine osmolality was assessed using an Advanced Osmometer (Advanced Instruments, Norwood, MA, USA). Urine specific gravity was determined with Siemens SG 10 dipsticks and analyzed using a Siemens Clinitek 500 analyzer. Urine color was compared with a validated urine color chart that has 8 color bands and assigns a numerical value ranging from 1 (lightest) to 8 (darkest) [13]. Urine color scores of 1 or 2 indicate that the subject is well hydrated, scores of 3 or 4 represent minimal dehydration, scores of 5 or 6 indicate significant dehydration, and scores of > 6 through 8 indicate serious dehydration.

### Statistical analyses

The protocol called for about 70 subjects to be screened, approximately 55 randomized, and 50 to complete the study. The primary analysis was performed on evaluable subjects (ie, subjects who did not violate the concomitant medications and lifestyle restrictions).

As this study was exploratory, only descriptive statistics were used with no criteria set for statistical or clinical significance. Shift tables were developed to display the shift in symptoms between Visit 1 (with URTI) and Visit 2 (14 to 21 days later, after recovery).

## Results

### Participants

Out of 58 subjects screened, 55 were enrolled (safety population), and 50 completed the study. Four subjects were lost to follow up and 1 failed to complete the study for an unknown reason. Subjects were mostly female (63.6%) and white (94.5%), and had a median age of 19 years (range 18 to 58 years); baseline demographics are shown in more detail in Table 2.

**Table 2:** Baseline demographics of the overall safety population (*N* = 55)

Characteristic	Participants
Sex, n (%)	
Female	35 (63.6)
Male	20 (36.4)
Race, n (%)	
White	52 (94.5)
Black	2 (3.6)
Asian	1 (1.8)
Age, years	
Mean (SD)	21.0 (6.84)
Median (range)	19.0 (18–58)

*SD* standard deviation

By Visit 2, URTI symptoms had largely resolved: 17% of subjects still had a mildly runny nose, 6% still had mild congestion, 6% still felt mildly tired, and 2% still felt mildly chilly. None of the subjects had any moderate or severe URTI symptoms, and none were still sneezing.

### Serum parameters of dehydration

Serum osmolality (the primary endpoint) and other blood parameters did not show evidence of dehydration during URTI (Table 3). A single subject experienced a shift in serum osmolality from normal during URTI to > 1.05 x upper limit of normal (ULN) after recovery.

**Table 3:** Summary statistics for serum parameters in evaluable subjects (*N* = 52^a^)

Serum Parameter	Visit 1 (With URTI)	Visit 2 (After Recovery)
Osmolality, mosm/kg, n	47	47
Mean (SD)	287.63 (4.83)	288.60 (5.99)
Median (range)	287.00 (278.0–307.0)	288.00 (278.0–319.0)
Complete blood count, n	48	48
Hemoglobin, g/L		
Mean (SD)	140.71 (12.53)	140.52 (14.07)
Median (range)	140.50 (118.0–167.0)	140.50 (111.0–176.0)
RBC count, × 10^12^/L		
Mean (SD)	4.78 (0.43)	4.78 (0.47)
Median (range)	4.84 (4.0–5.7)	4.77 (3.9–5.9)
Hematocrit, volume fraction		
Mean (SD)	0.43 (0.035)	0.43 (0.040)
Median (range)	0.43 (0.4–0.5)	0.43 (0.3–0.5)
Mean cell volume, fL/RBC		
Mean (SD)	89.98 (4.30)	89.97 (4.089)
Median (range)	90.60 (79.1–98.5)	90.65 (77.8–97.5)
Mean cell hemoglobin, pg/RBC		
Mean (SD)	29.45 (1.64)	29.41 (1.44)
Median (range)	29.75 (25.0–32.7)	29.60 (25.2–32.1)
Mean cell hemoglobin concentration, g/L		
Mean (SD)	327.29 (9.66)	326.92 (7.85)
Median (range)	325.50 (311.0–359.0)	326.00 (311.0–343.0)
White blood cell count, × 10^9^/L		
Mean (SD)	6.34 (1.81)	5.82 (1.33)
Median (range)	5.90 (2.9–10.5)	5.50 (3.3–10.9)
Platelet count, × 10^9^/L		
Mean (SD)	268.35 (57.43)	283.58 (63.89)
Median (range)	265.00 (160.0–428.0)	276.50 (170.0–466.0)
Serum urea, mmol/L, n	48	48
Mean (SD)	4.06 (0.908)	4.21 (1.097)
Median (range)	4.05 (2.0–6.1)	4.15 (2.0–8.1)
Serum electrolytes, n	48^b^	48^b^
Sodium, mmol/L		
Mean (SD)	140.40 (1.75)	140.70 (2.31)
Median (range)	140.60 (135.1–145.1)	140.65 (136.3–145.7)
Potassium, mmol/L		
Mean (SD)	4.22 (0.27)	4.19 (0.33)
Median (range)	4.20 (3.4–5.2)	4.21 (3.5–4.9)
Bicarbonate, mmol/L		
Mean (SD)	24.06 (2.26)	23.95 (2.31)
Median (range)	24.25 (18.5–29.5)	24.25 (18.4–29.6)
Chloride, mmol/L		
Mean (SD)	102.17 (2.36)	102.57 (2.59)
Median (range)	102.35 (94.6–107.2)	103.20 (95.4–107.4)

^a^Analyses included only subjects with assessments at both visits

^b^*n* = 47 for potassium

*RBC* red blood cell, *SD* standard deviation, *URTI* upper respiratory tract infection

Complete blood count measures largely remained stable during and after URTI, with a few exceptions. Two subjects had increases in hematocrit from normal during URTI to > 1.05 x ULN after recovery. Two subjects had decreases in hemoglobin, shifting from normal during URTI to < 0.95 x lower limit of normal (LLN) after recovery. One subject had an increase in mean cell hemoglobin, shifting from < 0.95 x LLN during URTI to normal after recovery. One subject’s white blood cell count shifted from normal during URTI to < 0.90 x LLN after recovery. One subject had an increase in platelet counts from normal during URTI to > 1.10 x ULN after recovery. All subjects had normal red blood cell counts and mean cell volume at both visits.

Two subjects had shifts in serum urea. One decreased from normal during URTI to < 0.90 x LLN after recovery, and the other increased from < 0.90 x LLN during URTI to normal after recovery.

Serum electrolytes largely remained stable during the study, with a few exceptions. Inconsistent changes in bicarbonate were observed, with 5 subjects showing a decrease from normal during URTI to < 0.95 x LLN after recovery, and 5 showing an increase from < 0.95 x LLN during URTI to normal after recovery. In 2 subjects, potassium levels decreased from normal during URTI to < 0.95 x LLN after recovery. All subjects had normal sodium and chloride levels at both visits.

### Urine parameters of dehydration

There were no changes in urine specific gravity or urine color [13] between Visits 1 and 2 (Table 4). Urine osmolality showed some variability. More subjects had increased urine osmolality (> 1.10 x ULN) during URTI (*n* = 31) than after recovery (*n* = 24), resulting in a higher overall population mean and median urine osmolality during URTI (Table 4). Fourteen subjects experienced a decrease in urine osmolality, shifting from > 1.10 x ULN to either normal (*n* = 9) or < 0.90 x LLN (*n* = 3), or shifting from normal to < 0.90 x LLN (*n* = 2). Five subjects experienced an increase in urine osmolality from within the normal range to > 1.10 x ULN, and the remaining 29 subjects experienced no categorical shifts in urine osmolality.

**Table 4:** Summary statistics for urine parameters in evaluable subjects (*N* = 52^a^)

Urine Parameter	Visit 1 (With URTI)	Visit 2 (After Recovery)
Specific gravity, n	48	48
Mean (SD)	1.02 (0.007)	1.02 (0.009)
Median (range)	1.02 (1.0–1.0)	1.02 (1.0–1.0)
Osmolality, mosm/kg of water, n	48	48
Mean (SD)	700.50 (231.59)	618.47 (320.29)
Median (range)	724.00 (149.0–1146.0)	632.50 (93.0–1222.0)
Color, points^b^, n	46	46
Mean (SD)	2.22 (0.89)	2.13 (1.05)
Median (range)	2.00 (1.0–4.0)	2.00 (1.0–5.0)

^a^Analyses included only subjects with assessments at both visits

^b^Urine color was matched to a validated color chart with 8 color bands, categorized as 1 (lightest) to 8 (darkest) points [13]

*SD* standard deviation, *URTI* upper respiratory tract infection

### Body weight and BMI

Out of 47 subjects with data from both visits, 23 (48.9%) had stable body weight at both visits; 16 (34.0%) lost > 1% of body weight, and 8 (17.0%) gained > 1.0% between the two visits (Table 5). Identical numbers of subjects exhibited those shifts in BMI (Table 5).

**Table 5:** Summary statistics for body weight and BMI in evaluable subjects (*N* = 47)

Parameter	Visit 1 (With URTI)	Visit 2 (After Recovery)
Body weight, kg		
Mean (SD)	71.63 (12.67)	71.13 (12.82)
Median (range)	71.0 (48.9–104.0)	71.6 (48.8–106.0)
BMI, kg/m^2^		
Mean (SD)	24.83 (3.75)	24.66 (3.86)
Median (range)	24.14 (19.5–38.7)	23.92 (19.3–39.4)

*BMI* body mass index, *SD* standard deviation, *URTI* upper respiratory tract infection

### Subjective assessments

**Fig. 1:**
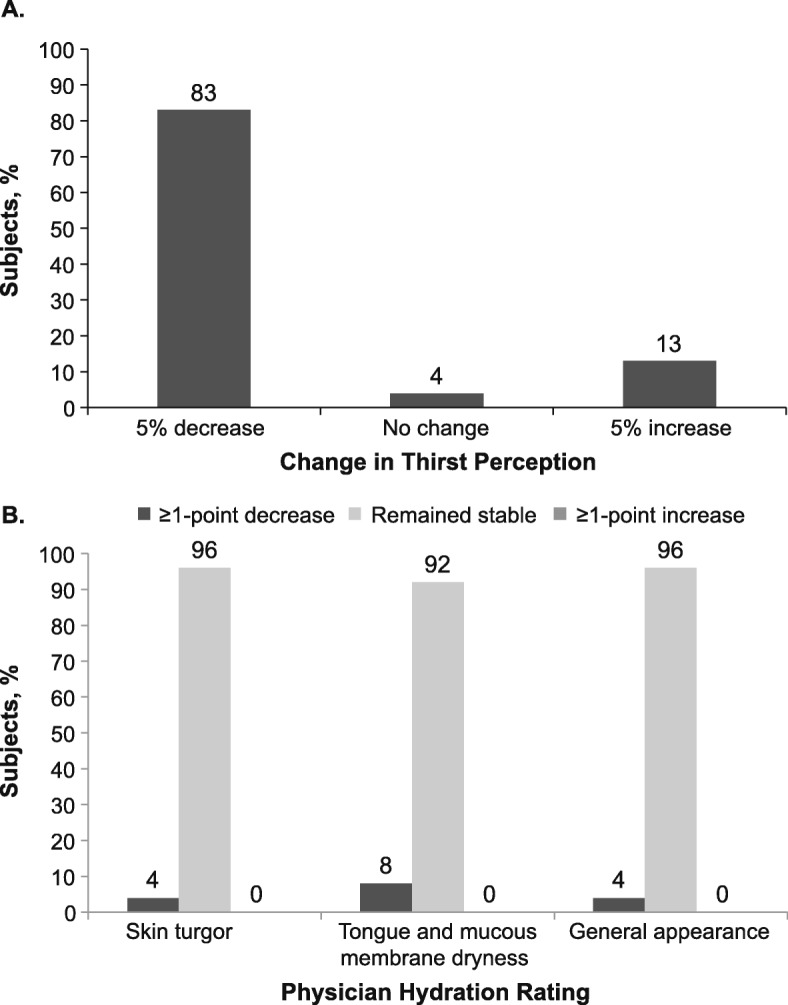
Shifts in (**a**) subject ratings of thirst^a^ and (**b**) physician ratings of hydration^b^ from visit 1 (with URTI) to visit 2 (after recovery) (*N* = 48). ^a^Subject ratings of thirst were based on a visual analog scale ranging from 0 = no thirst at all to 100 = worst thirst ever experienced. ^b^Physicians rated hydration on a scale of 0 = no evidence of dehydration, 1 = some dehydration, 2 = moderate dehydration, and 3 = severe dehydration. URTI, upper respiratory tract infection

Ratings of thirst based on the 100-mm VAS suggested that participants perceived greater thirst during URTI (Table 6) and that excess thirst generally abated after recovery (Fig. 1a). Physician assessments of hydration (Table 6) suggested that a majority of subjects (≥92%) had stable levels of hydration from Visit 1 to Visit 2 (Fig. 1b).

**Table 6:** Summary statistics for subject ratings of thirst and physician ratings of hydration (*N* = 48)

Parameter	Visit 1 (With URTI)	Visit 2 (After Recovery)
Subject ratings of thirst^a^		
Mean (SD)	44.17 (19.01)	17.17 (13.83)
Median (range)	46.50 (9.0–91.0)	17.00 (0.0–55.0)
Physician ratings of hydration^b^		
Skin turgor		
Mean (SD)	0.46 (0.58)	0.08 (0.28)
Median (range)	0 (0–2)	0 (0–1)
Tongue and membrane dryness		
Mean (SD)	0.85 (0.62)	0.10 (0.309)
Median (range)	1 (0–2)	0 (0–1)
General appearance		
Mean (SD)	0.42 (0.58)	0.08 (0.28)
Median (range)	0 (0–2)	0 (0–1)

^a^Subject ratings of thirst were based on a visual analog scale ranging from 0 = no thirst at all to 100 = worst thirst ever experienced

^b^Physicians rated hydration on a scale of 0 = no evidence of dehydration, 1 = some dehydration, 2 = moderate dehydration, and 3 = severe dehydration

*SD* standard deviation, *URTI* upper respiratory tract infection

### Safety

As expected, there were no reported adverse events in this non-interventional study.

## Discussion

Contrary to popular belief, URTIs did not lead to clinically meaningful signs of dehydration in our study. Greater urine osmolality and increased thirst during URTI were observed in some subjects, but serum parameters and physician ratings of hydration showed no clinically meaningful differences during URTI versus after recovery.

Plasma/serum osmolality is the most widely used hematologic measure of hydration status [14] and is considered by some experts to be the only valid marker of dehydration in an individual at a single time point [15]. Serum or plasma osmolality is tightly controlled with a set point of 280 to 290 mosm/kg that is stable within about ±2% among well-hydrated persons [12, 14, 16]. In contrast, during periods of fluid restriction, serum osmolality has been found to increase linearly, rising from 285 mosm/kg at baseline to 295 mosm/kg during normal activities [17] and from 295 to 303 mosm/kg during 60 min of cycling among subjects in a hypohydrated state (24-h fluid restriction) [18].

Even in studies where water was not restricted, increases in plasma osmolality have been observed during exercise. In 1 study, when water was consumed ad libitum during a half-marathon by moderately trained runners, plasma osmolality increased from a mean of 289 mosm/kg before the race to 296 mosm/kg afterward (*P* < 0.01) [19]. In another, serum osmolality increased from 291 to 298 mosm/kg during 60 min of cycling among euhydrated subjects [18].

The exact serum osmolality threshold for identifying dehydration remains controversial. Based on assessments in healthy male athletes, Armstrong et al. proposed that for a 75.1-kg man, a morning serum osmolality of ≥292 mosm/kg would be indicative of dehydration [20], whereas Cheuvront et al. proposed 301 mmol/kg as a threshold for identifying dehydration [15]. Mean and median serum osmolality in our study population was 287–288 mosm/kg both during and after URTI, a level consistent with being well hydrated and within the normal physiologic set point range [12, 20].

While 14 subjects in the current study had greater urine osmolality (ie, more concentrated urine) during URTI than after recovery, and mean urine osmolality was higher during URTI compared with after recovery (700.5 vs 618.5 mosm/kg), these results must be interpreted with caution, for numerous reasons. Urine osmolality findings were variable: 5 other subjects had decreased urine osmolality (ie, less concentrated urine) during URTI compared with after recovery. There is a lack of agreement as to what constitutes optimal urine osmolality and what the threshold should be for identifying dehydration [20–23]. In addition, these changes in urine osmolality were not accompanied by changes in serum osmolality (a more accurate marker of dehydration [19]). A recent investigation conducted in collegiate athletes found that use of urine-based definitions of dehydration (osmolality ≥700 mosm/kg H_2_O or urine specific gravity > 1.020) alone would have identified 27 to 55% of the athletes as dehydrated and in need of more fluids, whereas none of these athletes were confirmed to be dehydrated and in need of fluids based on serum Na + measurement [24].

Another reason for caution in interpreting the urine osmolality results is that urine osmolality is affected by the amount of fluid and food people consume as well as by kidney function, metabolism, fluid regulatory hormones, and kidney water conservation (which is a normal physiologic response to water deprivation that allows for maintenance of natremia) [12, 22, 24]. Fluid intake was not restricted or assessed during the current study, and individual participants may have either decreased their fluid intake due to loss of appetite during URTI, or increased their fluid intake due to increased thirst during URTI, which potentially represents a natural mechanism to prevent dehydration. It is also possible that some subjects increased their fluid intake to prevent dehydration, based on established folklore about risk of dehydration during URTI or recommendations from reputable medical institutions [1, 2]. Furthermore, sweating and other illness-related reasons for flux in body water can lead to inaccurate osmolality results especially when spot urine samples are used, as these tend to be highly variable [22, 25].

Some subjects exhibited shifts in body weight—including both gains and losses. These results cannot be interpreted with regard to hydration status given that they were made over an interval of 2 to 3 weeks, and change in adipose tissue during that time is unknown [14].

One of the most relevant differences observed was in perception of thirst: 83% of subjects reported at least a 5% increase in thirst while symptomatic. In general, thirst is prompted when total body water loss is > 2% [26]. However, this finding also should be interpreted with caution; while it may indeed be a function of a natural mechanism to maintain hydration during an illness, it could also be influenced by participant expectation of being more thirsty during URTIs, or be a result of increased mouth breathing due to nasal stuffiness, which can lead to dry mouth, which in turn creates a sensation of thirst. The latter explanation is in line with the 8-fold elevation in mean tongue and membrane dryness seen at Visit 1 compared to Visit 2 following recovery. Regardless, all of the subject and investigator ratings of thirst and hydration were higher during URTIs than after recovery, which could be indicative of thirst as a stimulus to prevent further dehydration in subjects with URTIs.

Contrary to the recommendation of Guy Vise, MD, in the late 1950s to induce dehydration as a means of relieving congestion during the common cold [27], current conventional wisdom is that people should drink extra fluids during acute URTIs such as common cold to prevent or treat dehydration, a recommendation that is endorsed by reputable medical establishments globally, such as the Mayo Clinic and NHS [1, 2]. Some physicians also recommend this approach as a strategy to thin and loosen respiratory secretions/mucus [28, 29].

However, increased fluid consumption may carry extra risks without evidence of benefit. A Cochrane meta-analysis that attempted to answer the question of whether increasing fluid consumption is safe and has benefit with regard to reduction in duration or severity of URTI symptoms ended up excluding all 166 records they reviewed, concluding that there are still no adequate randomized controlled trials to address this issue [29]. Cases have been reported in which overhydrating resulted in acute symptomatic hyponatremia (“water intoxication”), which in severe cases can be fatal [30, 31]. For this reason, the American Academy of Family Physicians advises against extra fluid intake for children with URTIs and notes that there is a lack of data to support this approach in adults [28].

This was a small pilot study; however, results may be informative given that there are no other published data in this area. The lack of control or assessment of fluid intake is also a limitation, as is the lack of virology information; however, these circumstances reflect real-world settings, in which subjects self-diagnose URTI based on symptoms and commonly self-treat and self-regulate fluid intake. Another limitation is that many hematologic and urinary measures of dehydration are not reliable when used over intervals of days to weeks [14]; however, a 2- to 3-week interval was necessary in the current study to allow adequate time for recovery from URTI before reassessment. Other limitations include the potential for inadequate representation of dynamic water turnover by a single measure in time (postbaseline) due to the constant fluctuations between fluid compartments [10] and the potential lack of sensitivity of current laboratory tests to assess the relatively minor imbalance in URTI-induced hydration status as compared with more severe illnesses such as hemorrhagic shock or cardiac failure [10, 11]. Furthermore, the mean age of the population in our current study is likely much younger than that in traditional hydration studies of acute or severe illnesses; however, as reported in a study of hydration in college athletes (ie, subjects closer in age to those in the current study), both plasma and urine osmolality were used to assess dehydration with only changes in urine osmolality detected, similar to our current study.

## Conclusions

In conclusion, the results of this pilot observational study found no evidence that URTIs such as common cold are associated with increased risk of dehydration, contrary to popular belief. However, participants were not required to have fever at the start of the study or as an inclusion criterion, so the results do not rule out the potential for dehydration during febrile illness associated with URTIs. Given that this is the first study to evaluate this question, additional studies are needed to confirm these findings and build a body of evidence.

## Data Availability

Anonymized individual participant data and study documents can be requested for further research from www.clinicalstudydatarequest.com.

## Abbreviations

BMI: Body mass index
LLN: Lower limit of normal
RBC: Red blood cell
SD: Standard deviation
ULN: Upper limit of normal
URTI: Upper respiratory tract infection
VAS: Visual analog scale
